# Dissecting Brainstem Locomotor Circuits: Converging Evidence for Cuneiform Nucleus Stimulation

**DOI:** 10.3389/fnsys.2020.00064

**Published:** 2020-08-21

**Authors:** Stephano J. Chang, Iahn Cajigas, Ioan Opris, James D. Guest, Brian R. Noga

**Affiliations:** ^1^Neuroscience Graduate Program, University of Miami Miller School of Medicine, Miami, FL, United States; ^2^The Miami Project to Cure Paralysis, University of Miami Miller School of Medicine, Miami, FL, United States; ^3^Division of Neurosurgery, Department of Surgery, University of British Columbia, Vancouver, BC, Canada; ^4^Department of Neurological Surgery, University of Miami Miller School of Medicine, Miami, FL, United States

**Keywords:** mesencephalic locomotor region, deep brain stimulation, gait dysfunction, cuneiform nucleus, pedunculopontine nucleus

## Abstract

There are a pressing and unmet need for effective therapies for freezing of gait (FOG) and other neurological gait disorders. Deep brain stimulation (DBS) of a midbrain target known as the pedunculopontine nucleus (PPN) was proposed as a potential treatment based on its postulated involvement in locomotor control as part of the mesencephalic locomotor region (MLR). However, DBS trials fell short of expectations, leading many clinicians to abandon this strategy. Here, we discuss the potential reasons for this failure and review recent clinical data along with preclinical optogenetics evidence to argue that another nearby nucleus, the cuneiform nucleus (CnF), may be a superior target.

## Introduction

Gait disturbances present in many neurological diseases and injuries, including Parkinson’s disease (PD), stroke, and spinal cord injuries. Neurological gait disorders are particularly common in older adults, with a prevalence of more than 20% after the age of 60 (Mahlknecht et al., 2013), and are likely to represent an increasing societal health burden as demographic shifts continue. These impairments lead to immobility and falls, and contribute to social isolation, reduced quality of life, and loss of independence (Mahlknecht et al., 2013). Few treatment options exist, making research in this field imperative. In this Perspective article, we review the preclinical developments that led to clinical trials for deep brain stimulation (DBS) of the pedunculopontine nucleus (PPN), discuss potential reasons why these trials have not been successful, and present new research supporting our view that the nearby cuneiform nucleus (CnF) may be a more efficacious target.

## The Mesencephalic Locomotor Region

**Figure 1:**
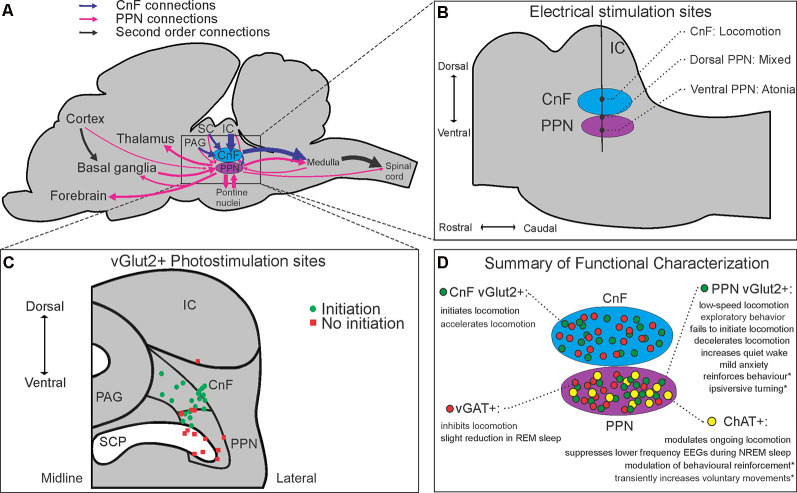
Anatomical, neurochemical, and connectome-based characterizations of the mesencephalic locomotor region (MLR). Schematic summaries of **(A)** major inputs and outputs of the CnF and PPN in rodents, **(B)** anatomical electrical mapping of the MLR in cats from Takakusaki et al. (2016); **(C)** CnF and PPN vGlut2+ photostimulation results from Josset et al. (2018) and **(D)** cell-type-specific functional characterizations of the CnF and PPN reported in the literature. Abbreviations: ChAT, choline acetyltransferase; CnF, cuneiform nucleus; IC, inferior colliculus; PAG, periaqueductal gray; PPN, pedunculopontine nucleus; SC, superior colliculus; SCP, superior cerebellar peduncle; vGAT, vesicular GABA transporter; vGlut2, vesicular glutamate transporter 2. *Denotes behavioral effects elicited from subsets of a neurochemical population projecting to specific targets.

The mesencephalic locomotor region (MLR) is a physiologically defined midbrain area, where low-threshold electrical stimulation initiates locomotion in decerebrate and intact animals (Shik et al., 1966; Mori et al., 1989). First described in cats in 1966, the MLR has since been identified as a conserved regulatory node within the supraspinal locomotor network in multiple vertebrate species (Eidelberg et al., 1981; Skinner and Garcia-Rill, 1984; Cabelguen et al., 2003; Ryczko and Dubuc, 2013), with electrophysiological and functional imaging evidence supporting its existence in humans (Jahn et al., 2008; Piallat et al., 2009). Anatomically, the MLR occupies the upper brainstem tegmentum, where it is hypothesized to integrate numerous sensorimotor, cognitive, and limbic inputs to regulate locomotion both directly, through descending reticulospinal and monoaminergic pathways to spinal locomotor networks (Noga et al., 2003, 2017b; Ryczko and Dubuc, 2013), and indirectly, through ascending connections to numerous higher brain centers (Figure 1A; Martinez-Gonzalez et al., 2011; Kroeger et al., 2017; Sébille et al., 2017). These diffuse projections also allow the MLR to regulate attention, arousal, and cortical state and couple them to locomotor states (Lee et al., 2014).

Historically, two adjacent nuclei have been put forth as putative neuroanatomical correlates of the MLR. Much of the preclinical literature, including Shik et al.’s original description (Shik et al., 1966), has supported the more dorsally located CnF, where electrical mapping studies consistently show it to promote locomotion (Figure 1B; Takakusaki et al., 2016; Opris et al., 2019). Conversely, others have favored the more ventral, cholinergic cell-containing PPN (Garcia-Rill et al., 1987), despite its more varied electrical mapping results (Figure 1B; Takakusaki et al., 2016).

Notwithstanding this controversy, two other converging narratives at the turn of the century ultimately led clinician-scientists to conduct non-human primate (NHP) PPN experiments: first, the discovery that dense inhibitory outputs from the globus pallidus interna terminated near the PPN generated the hypothesis that hyperactivity of the globus pallidus interna in PD could produce akinesia through excessive inhibition of the PPN (Aziz et al., 1998); and second, histopathological observations of the PPN in PD and progressive supranuclear palsy (PSP) patients showed significant cholinergic neuronal degeneration, suggesting a pathophysiological link to the dopamine-resistant postural instability and freezing of gait (FOG) commonly observed in these patients (Hirsch et al., 1987; Jellinger, 1988). Early NHP studies creating PPN area lesions did demonstrate significant akinesia (Aziz et al., 1998; Jenkinson et al., 2004), but received criticism for producing large lesions that may have encompassed surrounding structures (Winn, 2008). In response to these criticisms, Karachi et al. (2010) created cholinergic cell-specific lesions within the PPN of macaques that produced gait and balance abnormalities. While this seemed to provide evidence that cholinergic PPN neurons were critical for gait and posture, the urotensin II-conjugated toxin used in their study has only been shown to have selective effects on cholinergic cells of the PPN in rodents. The Supplemental data in Karachi et al.’s study demonstrates that the injected toxin also significantly destroyed noncholinergic cells in the region, preventing them from ruling out the possibility that the observed gait and balance abnormalities may have been due to other cell populations, such as glutamatergic neurons (Karachi et al., 2010).

Despite these and other potential concerns, including one NHP study showing worsening of akinesia and tremor with PPN stimulation (Nandi et al., 2002), DBS studies moved forward in patients with PD and PSP, and have focused solely on the PPN (Mazzone et al., 2005; Stefani et al., 2007; Ferraye et al., 2010; Moro et al., 2010; Doshi et al., 2015; Mestre et al., 2016). While these studies have documented the general safety of targeting this brainstem region for DBS, suboptimal targeting of the MLR has been proposed as a possible explanation for the mixed outcomes and muted efficacy of these DBS studies compared to earlier preclinical studies (Thevathasan et al., 2018). In the sections that follow, we further explore this hypothesis through an appraisal of the most current literature investigating the functional organization of the MLR. Furthermore, we propose that these studies support our perspective that glutamatergic neurons within the CnF likely represent the neuroanatomic basis for the MLR.

## Neurochemical Segregation of Functions Within the MLR

At least three neurochemical populations are found in the MLR, with glutamatergic and GABAergic neurons dispersed throughout the CnF and PPN, while cholinergic neurons have traditionally delineated the PPN (Figure 1D; Martinez-Gonzalez et al., 2012; Roseberry et al., 2016; Sebille et al., 2019). As cholinergic neurons were its most obvious immunohistochemical feature, the PPN was long considered a primarily cholinergic nucleus, with some attributing cholinergic neurons central importance in locomotor control (Ryczko and Dubuc, 2013). Stereological estimates in the rat PPN have since shown that cholinergic neurons number the fewest of the three neuronal subtypes (25%), with glutamatergic neurons being the most abundant (43%), followed by GABAergic neurons (32%; Luquin et al., 2018). Topographically, glutamatergic and cholinergic populations are concentrated caudally in the rat PPN, while GABAergic neurons are concentrated rostrally (Mena-Segovia et al., 2009; Luquin et al., 2018). Similar topographical descriptions of populations in the CnF are lacking.

These neurochemical populations have also been characterized in the human MLR (Pienaar et al., 2013; Sebille et al., 2019). Interestingly, while the rostrocaudal distribution of GABAergic neurons was found to be similar between humans and rats, cholinergic neurons demonstrated a reverse topography, suggesting species differences (Sebille et al., 2019). Mapping the distribution of glutamatergic neurons in the human MLR remains an important goal to complete this dataset and could have implications for DBS targeting. Notably, whereas earlier studies highlighted the loss of cholinergic neurons within the PPN in PD or PSP patients as evidence of their involvement in dopamine-resistant signs such as FOG (Hirsch et al., 1987; Jellinger, 1988; Zweig et al., 1989), newer studies reveal that noncholinergic neurons in both the PPN and CnF also show significant degeneration in PD and PSP (Pienaar et al., 2013; Sebille et al., 2019).

Optogenetic studies in mice provide further insights into the functions of these neurochemical populations. Roseberry et al. (2016) demonstrated that photoactivation of glutamatergic neurons in the mouse MLR initiates and controls the speed of locomotion, while photoinhibition of these neurons in running animals arrests them. Photoactivation of GABAergic neurons in the MLR also stopped locomotion, at least in part through local inhibitory mechanisms (Roseberry et al., 2016). Three separate studies have shown that photoactivation of cholinergic neurons in the PPN is unable to initiate locomotion, though it may modulate speed in ongoing locomotion (Roseberry et al., 2016; Caggiano et al., 2018; Josset et al., 2018). Altogether, these studies dispute the long-debated view that cholinergic PPN neurons play a primary role in the initiation and control of locomotion, implicating glutamatergic neurons instead (Noga et al., 2017a; Albin et al., 2018).

## Anatomical Segregation of Glutamatergic MLR Function

Two recent studies compared the function of glutamatergic neurons in the CnF and the PPN (Caggiano et al., 2018; Josset et al., 2018), while others have characterized the function of glutamatergic PPN neurons (Kroeger et al., 2017; Yoo et al., 2017; Assous et al., 2019). In support of this distinction, Caggiano et al. (2018) demonstrated that glutamatergic neurons in the CnF and PPN have vastly different inputs and outputs. While glutamatergic CnF neurons receive most of their monosynaptic inputs from a few midbrain structures, such as the inferior colliculus (IC) and the periaqueductal gray (PAG), glutamatergic PPN neurons receive projections from many brain regions, including numerous brainstem nuclei, the basal ganglia, hypothalamus, and frontal cortex (Figure 1A). Glutamatergic CnF neurons were also found to have a more focused descending output, primarily restricted to the ventrocaudal medulla, while glutamatergic PPN neurons showed broad projections to pontine, medullary, and even upper cervical spinal cord regions (Caggiano et al., 2018), all in addition to their numerous known ascending projections (Figure 1A; Martinez-Gonzalez et al., 2011; Kroeger et al., 2017; Yoo et al., 2017; Assous et al., 2019).

Both Caggiano et al. (2018) and Josset et al. (2018) show that activation of glutamatergic CnF neurons is sufficient to initiate locomotion at short latencies; however, the two studies differ on the role of PPN glutamatergic neurons. In addition to demonstrating increased exploratory behavior, Caggiano et al. (2018) found that photoactivation of glutamatergic PPN neurons was able to induce low-speed locomotion from rest in a subset (46%) of trials, albeit with longer latencies (~1 s) and requiring higher frequency stimulation (50 Hz). In contrast, Josset et al. (2018) found that photoactivation of glutamatergic PPN neurons, particularly ventrally, could not initiate locomotion, and decelerated ongoing locomotion.

Several potential reasons could explain this discrepancy—there were methodological differences in viral transfection of channelrhodopsin between the two groups and the viral expression profile in the PPN in Josset et al. (2018) appears more restricted than in Caggiano et al. (2018). Furthermore, there were methodological differences in the stimulation of the PPN between the two groups: dorsal PPN optrode locations with varying pulse width and frequency of stimulation by Caggiano et al. (2018) compared to both dorsal and ventral PPN optrode locations with a more uniform 10 ms pulse width, 20 Hz stimulation protocol by Josset et al. (2018). Interestingly, in trials where Josset et al. (2018) used crossed transgenic mice expressing channelrhodopsin in all glutamatergic neurons, the more dorsally located PPN stimulations were able to initiate low-speed locomotion from rest, where they could not with more ventrally located PPN stimulations (Figure 1C), nor with the PPN-specific virally-transfected mice. At least one other group supports the view that activation of glutamatergic CnF neurons produces robust locomotion in mice, while activation of glutamatergic PPN neurons reduces locomotor activity, citing important differences between these subpopulations again in terms of their connectivity, but also in their intrinsic membrane properties (Dautan et al., 2020). Their finding that glutamatergic CnF neurons are mostly rapidly adapting and accommodating, while glutamatergic PPN neurons are more heterogeneous in their electrophysiologic properties, provides further insight into why experiments regarding the PPN have often provided mixed behavioral results (Dautan et al., 2020).

A third study evaluating PPN glutamatergic function in mice using chemogenetics also suggests that these neurons do not directly control locomotion. Kroeger et al. (2017) found that beyond significantly increasing wake time, activating glutamatergic PPN neurons had highly context-dependent results. In the unenriched home cage, animals mostly sat quietly awake, with decreased exploration and feeding compared to normal wake behavior, while the addition of a running wheel encouraged moderate running comparable to that seen during normal wake cycles (Kroeger et al., 2017). In an open field, glutamatergic PPN activation led to animals spending more time in corners, suggesting mild anxiety (Kroeger et al., 2017). Taken together, these studies suggest that while the locomotor contributions of glutamatergic PPN neurons cannot be completely excluded, they likely represent a more functionally heterogeneous population than glutamatergic CnF neurons, which have a clearer role in the initiation and control of gait.

## Connectome-Based Evaluations of MLR Function

Given the diffuse projections of glutamatergic and cholinergic PPN neurons, several groups have isolated the effects of selectively activating glutamatergic or cholinergic terminals projecting from the PPN to specific targets of interest. One of the earliest optogenetic studies in the MLR showed that the enhanced visual processing observed with MLR stimulation could largely be dissociated from its locomotor effects by selectively activating PPN terminals targeting cholinergic groups of the basal forebrain (Lee et al., 2014). Additionally, activation of glutamatergic projections from the PPN to dopaminergic neurons in the ventral tegmental area, an area known for its involvement in motivation and addiction, induces robust reinforcement of lever-pressing behavior in mice (Yoo et al., 2017). In contrast, activating cholinergic PPN projections to the ventral tegmental area only delays the extinction of trained lever-pressing behavior (Dautan et al., 2016). Finally, unilateral activation of glutamatergic PPN projections to the striatum was found to induce ipsiversive head-turning (Assous et al., 2019). Overall, these studies demonstrate the diversity of behaviors attributable to the PPN and conceivably explain why electrical, pharmacological, and even optogenetic manipulations of the PPN have produced such variable behavioral results. Such functional heterogeneity, while potentially providing the PPN with important regulatory properties, ultimately makes the PPN a poor candidate target for DBS to promote a specific function such as gait.

Conversely, most glutamatergic CnF neurons project to glutamatergic reticulospinal neurons in the medial reticular formation as well as monoaminergic neurons in the locus coeruleus (LC) and raphe nuclei, which form important descending pathways to spinal locomotor networks (Steeves and Jordan, 1980; Noga et al., 2017b). In the mouse, these glutamatergic reticulospinal neurons are necessary and sufficient for mediating MLR-evoked locomotion. Tracing studies have shown that, in addition to receiving presynaptic inputs directly from multiple brain regions [including the superior colliculus (SC), hypothalamus, PAG, deep cerebellar nuclei, red nucleus (RN), zona incerta, and motor cortex], these reticulospinal neurons receive input from both the CnF and the PPN, with more input from the CnF (Capelli et al., 2017). This is consistent with the idea that glutamatergic CnF neurons play a primary role in brainstem locomotor control, and highlight its potential as a DBS target.

## Clinical Considerations

The results of DBS in this region for gait dysfunction have been variable, although a recent review and meta-analyses suggest that PPN DBS may provide a small benefit concerning postural instability, falls, and FOG (Wang et al., 2016, 2017; Thevathasan et al., 2018). Several explanations have been proposed to account for the absence of a larger effect. First, there may be significant species differences in MLR function, particularly given our bipedal gait and the dominance of our corticospinal tracts (Alam et al., 2011). These differences may have relegated the functional role of the MLR in human gait, such that DBS of this region may not have the same effects in humans. Another possibility is that the stimulation of degenerated or abnormal neurons in the MLR, especially in the context of diseased basal ganglia, may have limited efficacy in compensating for lost function (Benarroch, 2013). A third scenario is that the neurons within the PPN do not represent the MLR and that optimizing the targeting of the MLR could improve its effectiveness.

**Figure 2:**
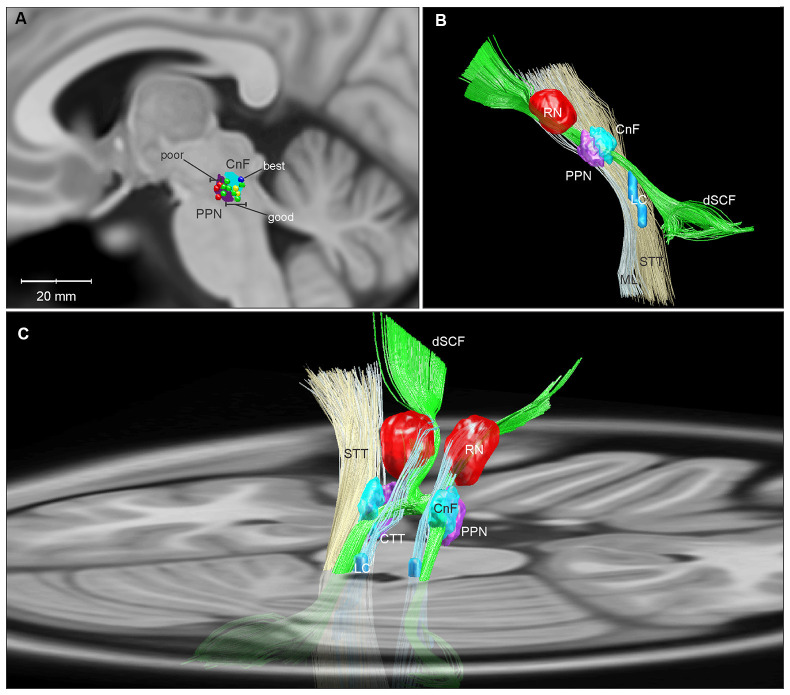
Three-dimensional reconstructions of the human MLR and regional anatomy. Reconstructions were made using Lead-DBS and available MNI-space subcortical atlases (Horn and Kühn, 2015). A separate CnF NIfTI object (cyan) was created in MATLAB in relation to the PPN (purple) based on Olszewski and Baxter (1982). **(A)** A parasagittal projection (5 mm lateral to the midline) of the CnF and PPN is overlaid with active contacts from PPN DBS patients with poor (red), good (green), best (dark blue), and unevaluated (yellow) FOG outcomes from Goetz et al. (2019). **(B)** Sideview of 3D reconstruction of MLR and regional anatomy, with left ML and STT removed to show the CnF and PPN. **(C)** Diagonal view with right ML and STT removed, projected on to a transverse slice of the brain at the level of the pons. Abbreviations: CnF, cuneiform nucleus; CTT, central tegmental tract; dSCF, decussating superior cerebellar fibers; LC, locus coeruleus; ML, medial lemniscus; PPN, pedunculopontine nucleus; RN, red nucleus; STT, spinothalamic tract.

Certainly, demonstrating success with DBS of the MLR would speak against the first two possibilities. A recent study of MLR DBS by Goetz et al. (2019) shows that DBS in this area can significantly alleviate FOG in PD patients and highlights the importance of electrode-position. Using responder analysis, the authors demonstrated that while there was significant variability in FOG outcomes among their subjects, categorizing the group by these outcomes revealed a “good responder” cluster with a significant reduction in % time spent in FOG with DBS on compared to off (34.1 ± 14% vs. 2.7 ± 2.6%). Furthermore, all of these good responders had active electrode contacts either in or bordering the CnF (Figure 2A; Goetz et al., 2019). This is in agreement with computer modeling studies of DBS in the region, which demonstrate that lead shifts as little as 1 mm significantly decrease target activation selectivity (Zitella et al., 2013).

The mechanisms of DBS, albeit incompletely understood, are currently believed to encompass electrical, cellular, molecular, and network effects on multiple timescales (Jakobs et al., 2019). Electrically, this is thought to include the induction of orthodromic and antidromic action potentials along both efferent and afferent fibers passing within an electrode’s volume of activation (Anderson et al., 2019; Jakobs et al., 2019). With regards to DBS of the MLR for gait, several potential mechanisms have been proposed: through ascending effects on arousal; by afferent-mediated disruption of cortical and subcortical pathological oscillations; through modulatory effects on other structures such as the subthalamic nucleus and substantia nigra; and through descending activation of spinal locomotor networks (Garcia-Rill et al., 2019). Based on the most current understanding of the MLR circuitry, and the anatomo-clinical findings in Goetz et al. (2019), our perspective is that DBS causes orthodromic activation of efferent and/or afferent fibers of glutamatergic CnF neurons targeting reticulospinal neurons in the medulla, which in turn excite spinal central pattern generators to promote gait (Noga et al., 2003).

## Conclusions and Future Directions

More than 50 years after the discovery of the MLR, and 15 years after the first-in-man reports of DBS of the PPN, there remains a conspicuous disconnect between basic science and clinical investigations into this midbrain region. Though the electrical mapping literature has arguably always favored the CnF, new insights into the functional organization of the MLR further challenges the exclusive focus on the PPN as a DBS target for enhancing gait. Several groups have started to acknowledge this in different ways, ranging from the adoption of a broader “PPN area” terminology (Ferraye et al., 2010; Mestre et al., 2016; Goetz et al., 2019), to asserting that DBS of the PPN should be reconsidered altogether (Albin et al., 2018). Given that subsets of PD patients demonstrate significant improvements in FOG with DBS in this region, further refinement of electrode location, stimulation parameters, and patient selection would seem a reasonable goal. Our view that the CnF should be considered as a DBS target is supported by studies suggesting that glutamatergic CnF neurons represent a primary locus for direct brainstem control of locomotion, with converging clinical evidence that dorsal locations within the MLR are associated with the best gait outcomes.

Currently, several labs are working to test the hypothesis that CnF DBS may improve gait function, including optogenetic studies assessing the contributions of glutamatergic CnF neurons to locomotor recovery in a rodent model of spinal cord injury (Roussel et al., 2019), detailed characterizations of CnF DBS in a large animal model (Chang et al., 2019), and a pilot clinical study of CnF DBS for FOG (Chang et al., 2020). Topographical analyses of glutamatergic projection neurons within the human CnF and MLR, including the location and orientations of their afferent and efferent pathways, may provide further guidance for targeting electrodes to optimally and selectively activating this circuit (Figures 2B,C).

## Data Availability Statement

The data analyzed in this study is subject to the following licenses/restrictions: the datasets generated during and/or analyzed during the current study are available from the corresponding author on reasonable request. Requests to access these datasets should be directed to bnoga@med.miami.edu.

## Author Contributions

All authors were involved in the conception and substantial revision of this manuscript. SC wrote the manuscript and drafted the figures.

## Conflict of Interest

The authors declare that the research was conducted in the absence of any commercial or financial relationships that could be construed as a potential conflict of interest.

## Abbreviations

CnF: cuneiform nucleus
DBS: deep brain stimulation
FOG: freezing of gait
MLR: mesencephalic locomotor region
NHP: non-human primate
PAG: periaqueductal gray
PD: Parkinson’s disease
PPN: pedunculopontine nucleus
PSP: progressive supranuclear palsy.
